# Reduced Pain and Inflammation in Juvenile and Adult Rats Fed a Ketogenic Diet

**DOI:** 10.1371/journal.pone.0008349

**Published:** 2009-12-23

**Authors:** David N. Ruskin, Masahito Kawamura, Susan A. Masino

**Affiliations:** 1 Department of Psychology and Neuroscience Program, Trinity College, Hartford, Connecticut, United States of America; 2 Department of Pharmacology, Jikei University School of Medicine, Minato-ku, Tokyo, Japan; AgroParisTech, France

## Abstract

The ketogenic diet is a high-fat, low-carbohydrate regimen that forces ketone-based rather than glucose-based cellular metabolism. Clinically, maintenance on a ketogenic diet has been proven effective in treating pediatric epilepsy and type II diabetes, and recent basic research provides evidence that ketogenic strategies offer promise in reducing brain injury. Cellular mechanisms hypothesized to be mobilized by ketone metabolism and underlying the success of ketogenic diet therapy, such as reduced reactive oxygen species and increased central adenosine, suggest that the ketolytic metabolism induced by the diet could reduce pain and inflammation. To test the effects of a ketone-based metabolism on pain and inflammation directly, we fed juvenile and adult rats a control diet (standard rodent chow) or ketogenic diet (79% fat) ad libitum for 3–4 weeks. We then quantified hindpaw thermal nociception as a pain measure and complete Freund's adjuvant-induced local hindpaw swelling and plasma extravasation (fluid movement from the vasculature) as inflammation measures. Independent of age, maintenance on a ketogenic diet reduced the peripheral inflammatory response significantly as measured by paw swelling and plasma extravasation. The ketogenic diet also induced significant thermal hypoalgesia independent of age, shown by increased hindpaw withdrawal latency in the hotplate nociception test. Anti-inflammatory and hypoalgesic diet effects were generally more robust in juveniles. The ketogenic diet elevated plasma ketones similarly in both age groups, but caused slowed body growth only in juveniles. These data suggest that applying a ketogenic diet or exploiting cellular mechanisms associated with ketone-based metabolism offers new therapeutic opportunities for controlling pain and peripheral inflammation, and that such a metabolic strategy may offer significant benefits for children and adults.

## Introduction

Pain and inflammation are hallmarks of diverse acute and chronic diseases. Chronic pain is one of the most commonly indicated health-related factors leading to poor quality of life [1], [2], and, across all cultures, patients with chronic pain have among the lowest reported quality-of-life scores of any medical condition [1], [2]. In parallel, accumulating evidence points to inflammation as not simply a consequence but an active contributor to pathologies such as atherosclerosis, stroke, metabolic syndrome and cancer [3]. Without question, a great unmet public health need exists for safe, effective and non-addictive strategies to reduce pain and inflammation.

Dietary therapy has long been coveted as a strategy to treat a variety of clinical conditions, including pain and inflammation. For example, polyunsaturated fatty acids reduce nociception by activating peroxisome proliferator-activated receptors (PPARs) [4], and olive oil polyphenolic compounds reduce experimental inflammation [5]. In addition to specialized dietary approaches, chronic caloric restriction reduces inflammation in several models [6], [7]. Benefits of metabolic therapy are demonstrated unequivocally in disorders of amino acid metabolism (such as phenylketonuria), familial hypercholesterolemia, and disorders of fatty acid transport and oxidation [8], [9]. Overall, metabolism has clear effects on the central nervous system and a host of peripheral tissues, and strategies that exploit broadly the therapeutic benefits of metabolism are becoming more compelling in translational and clinical research [10]–[12].

Evidence is building steadily on the effectiveness of a ketogenic diet – a high-fat, low-carbohydrate regimen – in treating epilepsy, brain cancer, type II diabetes and neurodegeneration [13]–[15]. For decades the ketogenic diet has been used successfully to treat epilepsy, particularly pediatric and medically refractory epilepsy, and its efficacy has been validated by a host of multi-center, retrospective and randomized, prospective clinical studies [13], [16], [17]. The restricted carbohydrate content of a ketogenic diet minimizes glucose metabolism and increases ketolysis, i.e., the use of ketone bodies (acetone, acetoacetate, β-hydroxybutyrate) as alternate energy sources. Established cellular consequences and recently hypothesized mechanisms of ketogenic diet therapy [18]–[21] coalesce to suggest that a predominantly ketone-based metabolism may reduce inflammation and nociception as compared to glucose-based metabolism [12].

To date, published data characterizing the relationship among ketogenic diets, pain and inflammation are limited. A pilot clinical study showed that a ketogenic diet reduced inflammation in non-alcoholic fatty liver disease [22], and a need for more research on this topic has been noted recently [12], [23]. Data characterizing ketogenic diets and pain are also limited [24], although the use of anticonvulsant drugs as antihyperalgesic/antiallodynic agents for neuropathic pain suggests that an anticonvulsant ketogenic diet might be effective in reducing pain. In the present study we evaluated the therapeutic potential of a ketogenic diet directly by quantifying standard measures of pain and inflammation in juvenile and adult rats. We found that maintenance on an ad libitum ketogenic diet for three weeks attenuates thermal nociception and decreases a peripheral inflammatory response significantly in both age groups. These results indicate that metabolism-based strategies may offer new therapeutic opportunities with broad clinical implications.

## Results

**Figure 1:**
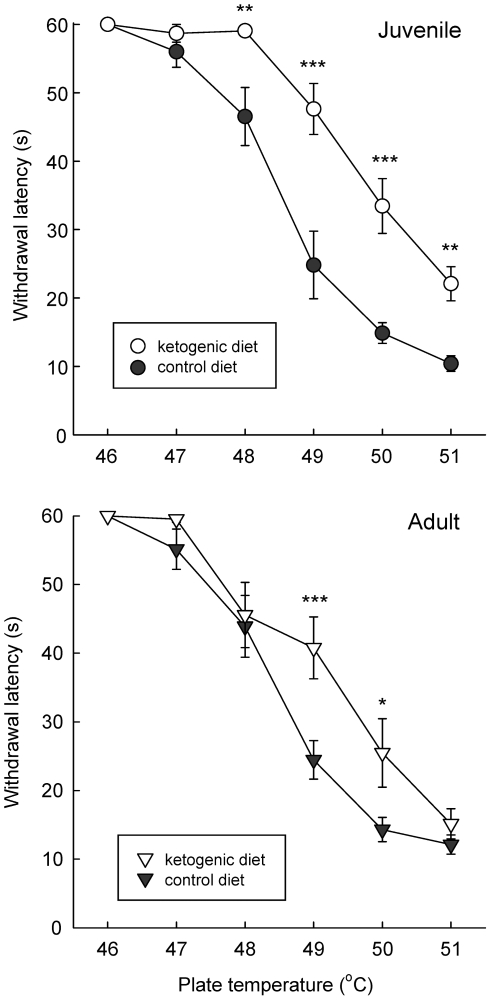
Hindpaw thermal nociception is decreased in juvenile and adult rats fed the ketogenic diet. *Top:* juvenile; *Bottom:* adult. All animals were tested on one temperature per day. Increasing hot plate temperature led to reduced withdrawal latency in all groups, and temperature-response curves were similar for juveniles and adults on the control diet (control diet – filled symbols, ketogenic diet – empty symbols). The ketogenic diet increased latencies in both age groups, with a more robust effect in juveniles. Analysis of juveniles revealed significant effects of temperature (F = 100.7, p<0.001) and diet (F = 18.9, p<0.001), and a significant diet x temperature interaction (F = 7.1, p<0.001). Analysis of adults revealed significant effects of temperature (F = 101.0, p<0.001) and diet (F = 4.8, p<0.05), and a significant diet x temperature interaction (F = 2.7, p<0.05). Numbers of subjects: 12 for each juvenile group, 14 for adult control diet, 16 for adult ketogenic diet. *p<0.05, **p<0.01, ***p<0.001; Newman-Keuls comparison to temperature-matched controls.

Latency to hindpaw withdrawal from a hotplate is a standard test for thermal nociception. All animals were tested with one temperature per day for six days (46–51°C). As expected, all diet and age groups exhibited a significant decrease in latency to hindpaw withdrawal as hotplate temperature increased (Figure 1). There was, however, notable hypoalgesia in the ketogenic diet-fed groups. Withdrawal latencies were significantly longer at temperatures 48–51°C in juveniles (Figure 1, top) and 49–50°C in adults (Figure 1, bottom). No significant differences were found at other temperatures. The diet effect appeared to be stronger in juveniles, with a larger rightward shift of the temperature-response curve and more highly significant post-hoc comparisons (Figure 1). Overall, maintenance on a ketogenic diet produced a clear hypoalgesic effect in juvenile and adult rats as assessed by hot plate testing.

**Figure 2:**
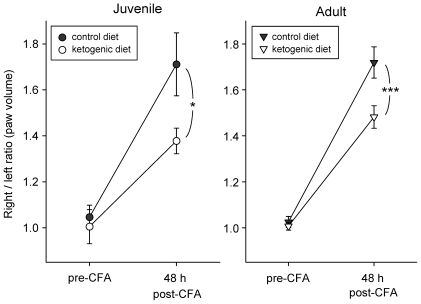
Hindpaw inflammatory swelling is reduced in juveniles and adults fed the ketogenic diet. Control diet – filled symbols, ketogenic diet – empty symbols for juveniles (*left*) and adults (*right*). Hindpaw volumes are shown as right/left ratios, measured just prior to and 48 h after injection of CFA into the right hindpaw. As expected, baseline ratios are similar to 1.0 in all groups. CFA-induced swelling was similar in juvenile and adult rats (71% and 72%, respectively), and was attenuated significantly by the ketogenic diet in both age groups. Analysis of juveniles indicated a significant effect of CFA (F = 68.7, p<0.001), and a significant diet x CFA interaction (F = 5.4, p<0.05). Analysis of adults indicated significant effects of CFA (F = 183.6, p<0.001) and a significant diet x CFA interaction (F = 7.5, p<0.05). Numbers of subjects: 7 for juvenile control diet, 6 for juvenile ketogenic diet, 12 for adult control diet, 11 for adult ketogenic diet. * p<0.05, *** p<0.001 Newman-Keuls comparisons to post-CFA controls.

We quantified peripheral inflammation in response to local injection of complete Freund's adjuvant (CFA). Just prior to CFA injection, hindpaw volumes of juvenile and adult rats maintained on a ketogenic versus control diet for 3 wk were measured. As expected, there was no significant right/left asymmetry in baseline hindpaw volume in any group and thus baseline right/left ratios were not different than 1.0 (Figure 2). Each animal received a CFA injection into the right hindpaw and we measured right/left hindpaw volume 48 h after CFA injection (peripheral inflammation peaks at approximately 48 h). The right/left ratio significant increased in all age and diet groups, indicating right hindpaw paw swelling. However, the ratio (and thus the injected paw volume) was significantly lower in both age groups fed the ketogenic diet (Figure 2). The swelling induced by CFA injection was similar in both age groups on the control diet, and the significant anti-inflammatory effect of the ketogenic diet was also similar in both age groups (Figure 2).

**Figure 3:**
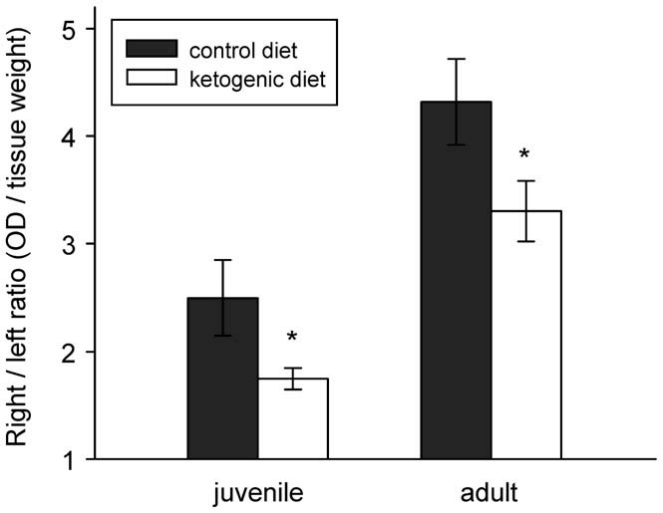
CFA-induced plasma extravasation is reduced in juvenile and adult rats fed the ketogenic diet. Control diet – solid bar, ketogenic diet – open bar for juveniles (*left*) and adults (*right*). Values are shown as right/left ratios and so would be near 1.0 for untreated animals. CFA-induced plasma extravasation was attenuated significantly by a ketogenic diet in juvenile and adult rats; CFA-induced plasma extravasation was lower overall in juveniles. *p<0.05, t-test comparisons to age-matched controls. Number of subjects: 5 for juvenile control diet, 6 for juvenile ketogenic diet, 10 for adult control diet, 11 for adult ketogenic diet.

To further characterize the peripheral inflammatory response, we quantified plasma extravasation, a measure of the movement of fluid to the extravascular space. Consistent with the reduced hindpaw swelling, ketogenic diet-fed rats of both ages had a significantly attenuated plasma extravasation response to CFA as compared to control diet-fed rats (Figure 3). The magnitude of the effect appeared to be larger in juveniles (extravasation attenuated by 50±7%) than in adults (attenuated by 31±8%).

**Figure 4:**
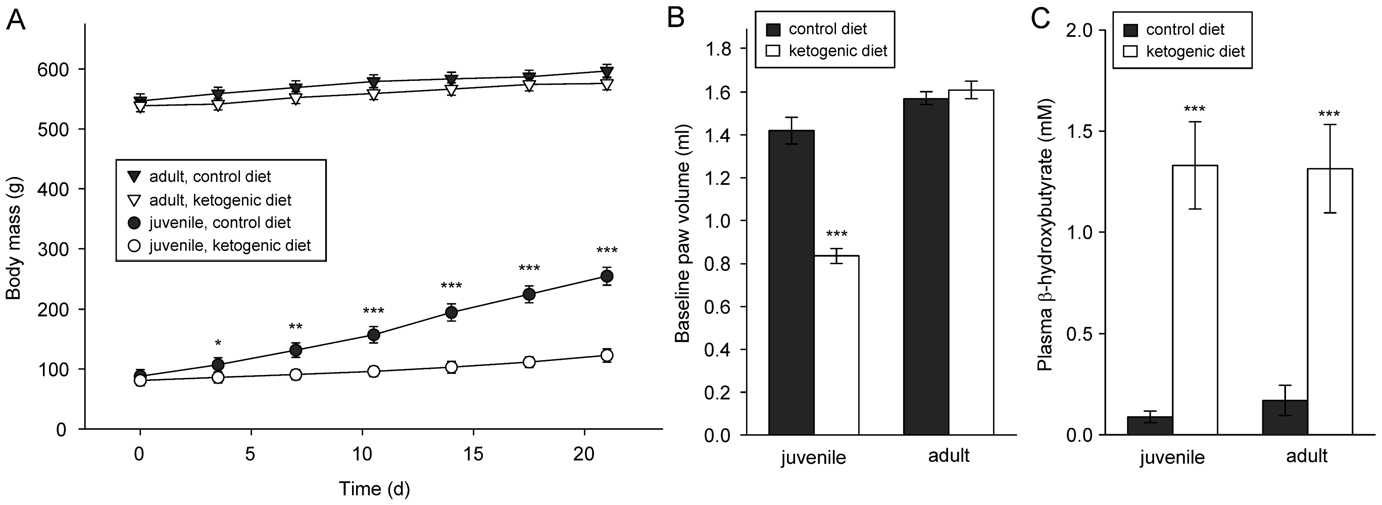
The ketogenic diet retards growth in juvenile but not adult rats, while inducing equivalent ketosis. *A:* All groups gained weight over three weeks. There was no difference in weight gain between diet groups in the adult animals; juvenile animals on the ketogenic diet gained weight significantly more slowly than those on the control diet. Analysis of juveniles revealed significant effects of time (F = 635.1, p<0.001) and diet (F = 17.6, p<0.001), as well as a significant time x diet interaction (F = 241.8, p<0.001), whereas for adults, there was a significant effect of time (F = 62.7, p<0.001) but not diet (F = 1.2, n.s.) and no significant interaction (F = 1.2, n.s.). Numbers of subjects: 12 for each juvenile group, 14 for adult control diet, 16 for adult ketogenic diet. *B:* Like body weight, baseline paw size (before CFA injection) was lower with the ketogenic diet in juveniles but not in adults. Numbers of subjects as in Fig. 2. *C:* Plasma levels of the ketone body β-hydroxybutyrate were similarly elevated in juvenile and adult rats. Analysis revealed a significant effect of diet (F = 49.6, p<0.001) but not age (F = 0.8, n.s.) and no significant interaction (F = 0.8, n.s.). Number of subjects = 7–9. *p<0.05, **p<0.01, ***p<0.001 Newman-Keuls (A,C) or t-test (B) comparisons to age-matched controls.

Even with ad libitum feeding, ketogenic diet-fed juveniles had significantly slower weight gain and growth rate as compared to juveniles on a control diet (Figure 4A), similar to clinical findings in pediatric epilepsy [25]; nevertheless, ketogenic diet-fed juveniles appeared healthy and active. Consistent with their lower body weight, ketogenic diet-fed juveniles had a significantly lower baseline hindpaw volume (Figure 4B) and so were injected with a proportional volume of CFA into the paw. In adults, there was no significant effect on weight (and no difference in hindpaw volume) between animals fed a ketogenic diet versus control diet (Figure 4A, B). Thus, hindpaw CFA injection volume was the same in all adults. The ketogenic diet increased blood ketones strongly and equivalently in juveniles and adults (Figure 4C). Therefore, differences in the level of ketosis do not account for the greater effects of the ketogenic diet in juvenile rats on some of the present measures.

## Discussion

Here we demonstrate hypoalgesic and anti-inflammatory effects of a ketogenic diet. In juvenile and adult rats we show that ad libitum feeding of a ketogenic diet reduces nociception, as assessed by hindpaw withdrawal latency, and peripheral inflammation, as assessed by CFA-induced hindpaw swelling and plasma extravasation. To date the clinical applications of ketogenic strategies have focused primarily on its established success with pediatric epilepsy [13] and emerging success with diabetes [26]; recent translational research is expanding clinical implications to include brain cancer, brain injury, and Rett syndrome [14], [15], [27]. New therapies are particularly urgent for pain, inflammation and inflammatory pain, and the present data suggest more translational research is needed for ketogenic diet therapy and analogous metabolic treatments.

There are a number of mechanisms thought to underlie the efficacy of ketogenic diet therapy, but an incomplete understanding of critical cellular mechanisms has hampered efforts to develop alternate pharmacological strategies and, in parallel, limited clinical predictions and applications of this type of metabolic therapy. However, published experimental research and hypotheses regarding the success of ketogenic diet therapy point to its clinical potential for pain and inflammation [18]–[21]. With respect to central pain mechanisms and neuronal activity, ketolytic metabolism is thought to increase levels of adenosine and/or GABA, two powerful inhibitory substances in the nervous system, through augmented oxidative phosphorylation and shifted glutamate:aspartate aminotransferase equilibrium, respectively [18], [28]. There is abundant evidence that increasing central inhibition by activating adenosine A_1_, GABA_A_ or GABA_B_ receptors produces hypoalgesia in acute pain tests [29], [30]. In addition to central mechanisms, a high polyunsaturated fatty acid content in ketogenic diets should enhance potassium conductances in peripheral neurons through PPAR activation [4], [31]. Therefore, we speculate that mechanistically-separate inhibitory processes in the central and peripheral nervous system could combine to mediate ketogenic diet-induced thermal hypoalgesia. Given the positive effects of adenosine and GABA agonists in treating chronic inflammatory and neuropathic pain [30], [32], [33] and the central hyperexcitability in chronic pain [34], ketogenic diets might be especially effective analgesics/hypoalgesics for diverse types of chronic pain. The success of dietary therapy even in pharmacoresistant epilepsy suggests that it may also be effective for intractable pain.

In addition to decreased nociception, we show that pretreatment with a ketogenic diet reduces subcutaneous inflammation significantly in juvenile and adult animals. There are multiple possible mechanisms. Ketone metabolism results in a decreased production of reactive oxygen species [35], [36], known to contribute to inflammation [37]. Adenosine acting through A_1_ and A_2_ receptor subtypes limits inflammation in a wide variety of peripheral and central tissues [38], [39], including inflammation due to subcutaneous inflammogens [30], [40]. Polyunsaturated fatty acid-induced PPAR activation inhibits NFκB and AP-1, both pro-inflammatory transcription factors [20]. It is possible that each is involved, and more research is needed to elucidate the primary mechanism underlying this peripheral effect. In addition to specific cellular mechanisms, overall protein restriction reduces inflammation in some situations [41], [42], and caloric restriction is anti-inflammatory in general [6], [7]. All animals in this study were fed ad libitum, thus it is unlikely that caloric restriction is occurring in the present study in the adult animals, a group that showed no difference in weight but did exhibit significantly reduced inflammation (and nociception) on the ketogenic diet. Nevertheless, a combined calorically-restrictive and ketogenic diet may be even more effective against inflammation (and potentially nociception) than either dietary component alone; similar conclusions have been made concerning the anticonvulsive and anticancer effects of dietary treatments [14], [43]. Furthermore, although we used the ketogenic diet as a pretreatment, clinical evidence suggests that it can reduce pre-existing liver inflammation [22].

In the one published study of nociception and the ketogenic diet, Ziegler et al. [24] described decreased (rather than increased) tail-flick latency in rats fed a ketogenic diet. Though both the hotplate and tail-flick involve thermal nociception, this difference may be related to methodological differences including rat strain, body part tested, diet composition, stimulus strength (latencies are generally longer in our study), and length of dietary treatment (12 wk in Ziegler et al.). The length of treatment might be particularly important, as a number of studies using several different measures have demonstrated non-monotonic effects of ketogenic diets at time scales of days to weeks [44], [45]. These variables should be examined in future studies, along with other pain modalities.

Complementing our direct experimental evidence, multiple hypotheses regarding the mechanisms underlying the success of ketogenic diet therapy coalesce to suggest that this metabolic treatment will reduce pain and inflammation. Yet despite widespread interest in dietary therapies for pain and inflammation (and myriad diseases that implicate inflammation as either a cause or a consequence of the pathology) systematic study of ketogenic strategies as anti-inflammatory or hypoalgesic strategies is just beginning. Unlike myriad dietary regimens with limited or inconsistent proof-of-efficacy, a ketogenic diet offers recognized and established clinical benefits [13]. Accordingly, there is a focus on elucidating critical mechanisms underlying the success of ketogenic diet therapy in treating epilepsy as well as mechanisms underlying emerging benefits in clinical conditions such as diabetes, brain injury, brain cancer and Rett syndrome [12], [14], [26], [46], [47]. The data presented herein suggest that ketogenic diets offer promising therapeutic potential for diverse inflammatory or painful conditions, across age groups, without the added difficulty of maintaining caloric restriction. Based on these results and many decades of clinical experience with diet-based therapies for pediatric epilepsy, a novel anti-inflammatory and hypoalgesic application of ketogenic diet therapy (or an analogous future pharmacological strategy) would be effective, non-addictive and relatively free of major side effects.

## Materials and Methods

Male Sprague-Dawley rats were bred in the Trinity College vivarium with animals originally purchased from Charles River (Storrs Mansfield, Connecticut, USA). All experiments were carried out in accordance with the NIH Guide for the Care and Use of Laboratory Animals and with approval of the Trinity College animal care and use committee. Either shortly after weaning at 21 d or as adults (85–110 d), matched groups of male Sprague-Dawley rats were switched to a ketogenic diet (AIN-76 Modified, High fat, #3666; Bio-Serv, Frenchtown, New Jersey, USA) or maintained on their standard diet (Purina 5001; PharmaServ, Framingham, Massachusetts, USA). Sprague-Dawley rats become ketotic within 5 d of ad libitum feeding of this particular ketogenic diet [48]. All animals were weighed twice weekly until the start of testing. All animals appeared healthy and normally active during dietary treatment.

After 3 weeks, and during continuing dietary treatment, rats were tested on a hotplate (Columbus Instruments, Columbus, Ohio, USA) at each integer temperature between and including 46° and 51°C; one temperature was tested per day, in ascending order. Based on preliminary testing, this temperature range started at a temperature that rarely produced a nocifensive response within 60 s (46°C) and went up in integers to the temperature that produced a response by approximately 10 s in control diet animals (51°C). To quantify thermal nociception at each temperature, rats were placed on the hot plate and the latency recorded to hindpaw-associated nocifensive behavior, typically suspension of the hindpaw or hindpaw-directed licking. Once such signs were observed, animals were removed immediately. To prevent any tissue damage, rats that reached 60 s without a response were removed and scored as 60 s.

After 4 weeks of dietary treatment, rats received an intraplantar injection of CFA (a suspension of heat-killed Mycobacterium tuberculosis, undiluted) in the right hindpaw to induce a consistent and sustained local inflammation. Hindpaw sizes were measured by volume displacement and paralleled total body size (Figure 4, right); the amount of injected CFA was adjusted accordingly to give an equivalent dose per paw size. CFA injection volume ranged from 100 µl (juveniles on ketogenic diet) to 190 µl (adults). Hindpaw size was measured by volume displacement just before and at 48 h after injection, a time-point selected to approximate the maximal inflammatory response; diet treatments were continued during this 48 h interval.

After final volume measurements, the dye Evans Blue was injected intravenously (60 mg/kg in a tail vein) to assess plasma extravasation (fluid movement from the intra- to the extravascular space), a major component of the inflammatory response. Tail vein injections were unsuccessful in four rats, and these were excluded from analysis. Two h after injection, rats were sacrificed by anesthesia overdose. After allowing intravascular dye to drain, hindpaw tissue was soaked in formamide at room temperature for several days to leach out extravascular dye. Duplicate aliquots of formamide were measured for optical density at 630 nm to quantify the level of Evans Blue. For juveniles, the same rats were used for pain and inflammation studies (baseline paw measurements and CFA injections occurred 1 d after the last hot plate test); for adults, separate groups were used. In all cases, CFA injection occurred after approximately four weeks of dietary treatment.

In a separate cohort of animals, trunk blood was collected after 3.5–4 wk of dietary treatment to assess levels of circulating ketones. These animals were not injected with CFA or Evans blue. Plasma β-hydroxybutyrate was measured with a Precision Xtra monitor and ketone test strips (Abbott Laboratories; Abbott Park, Illinois, USA).

Chemicals and CFA were purchased from Sigma-Aldrich (St. Louis, Missouri, USA). Data were analyzed with unpaired t-test or two-way repeated-measures analysis of variance as appropriate. Data are presented as mean±standard error.
